# The IL-8/IL-8R Axis: A Double Agent in Tumor Immune Resistance

**DOI:** 10.3390/vaccines4030022

**Published:** 2016-06-24

**Authors:** Justin M. David, Charli Dominguez, Duane H. Hamilton, Claudia Palena

**Affiliations:** Laboratory of Tumor Immunology and Biology, Center for Cancer Research, National Cancer Institute, National Institutes of Health, Bethesda, MD 20892, USA; justin.david@nih.gov (J.M.D.); charli.dominguez@nih.gov (C.D.); hamiltondh@mail.nih.gov (D.H.H.)

**Keywords:** IL-8, CXCR1/2, EMT, neutrophil, MDSC, brachyury, immune resistance

## Abstract

Interleukin-8 (IL-8, CXCL8) is a pro-inflammatory chemokine produced by various cell types to recruit leukocytes to sites of infection or tissue injury. Acquisition of IL-8 and/or its receptors CXCR1 and CXCR2 are known to be a relatively common occurrence during tumor progression. Emerging research now indicates that paracrine signaling by tumor-derived IL-8 promotes the trafficking of neutrophils and myeloid-derived suppressor cells (MDSCs) into the tumor microenvironment, which have the ability to dampen anti-tumor immune responses. Furthermore, recent studies have also shown that IL-8 produced by the tumor mass can induce tumor cells to undergo the transdifferentiation process epithelial-to-mesenchymal transition (EMT) in which tumor cells shed their epithelial characteristics and acquire mesenchymal characteristics. EMT can increase metastatic dissemination, stemness, and intrinsic resistance, including to killing by cytotoxic immune cells. This review highlights the dual potential roles that the inflammatory cytokine IL-8 plays in promoting tumor resistance by enhancing the immunosuppressive microenvironment and activating EMT, and then discusses the potential for targeting the IL-8/IL-8 receptor axis to combat these various resistance mechanisms.

## 1. Introduction

In order to grow and spread beyond the confines of a primary tumor, carcinoma cells must accomplish many difficult tasks, including gaining motility, degrading the extracellular matrix, accessing the blood supply, and successfully completing metastatic colonization by thriving within tissue environments that differ from those of the primary tumor. Perhaps one of the most challenging barriers to the successful spread of cancer is the constant threat of recognition and destruction by the host immune system. An increasing body of evidence indicates that the immune system plays a vital role in monitoring and controlling tumor development and progression, and immune evasion has been recognized as an “emerging hallmark” of cancer [1]. Newly developed immunotherapy-based approaches to treating cancer have achieved remarkable clinical successes in recent years. However, cells of the innate and adaptive immune system that are poised to eliminate tumor cells can be stifled by various cellular and molecular mechanisms that subdue their activation and/or effector functions. Overcoming these resistance mechanisms will be necessary to realize the full potential of immunotherapy [2].

Tumor cells can acquire the expression of various cytokines and their receptors to exploit these molecules for their own use. Cytokines secreted by the tumor can act on the surrounding normal stroma, recruiting them to aid in the growth, survival, and spread of the tumor. Tumor cells may also benefit directly from cytokine signaling if they have gained the expression of the cognate cytokine receptors, thereby allowing them to activate autocrine positive feedback loops. One such cytokine/receptor pair is the interleukin-8/interleukin-8 receptors (IL-8/IL-8R). This cytokine axis can substantially alter leukocyte infiltration into the tumor, resulting in the accumulation of immunosuppressive and pro-tumorigenic immune cells that can provoke the dysfunction of cytotoxic antitumor immune cells. IL-8/IL-8R signaling can also modulate the phenotypic status of tumor cells by activating a cellular differentiation program known as epithelial-mesenchymal transition (EMT), which endows tumor cells with enhanced metastatic, stemness, and resistance qualities. This review highlights the dual role that the inflammatory cytokine IL-8 plays in promoting tumor resistance by enhancing the immunosuppressive microenvironment and activating EMT, and then discusses the potential for targeting the IL-8/IL-8R axis to combat these various resistance mechanisms.

## 2. The IL-8/IL-8R Axis in Inflammation and Tissue Injury

Chemokines are a family of cytokines that cause the directed migration of leukocytes along a concentration gradient, resulting in the accumulation of the migrating cells at the source of chemokine production. IL-8, also known as CXCL8, is a pro-inflammatory CXC chemokine that was discovered for its role in promoting chemotaxis and degranulation of neutrophils [3]. It signals via binding with the G protein-coupled receptors cysteine-X-cysteine chemokine receptor 1 (CXCR1, IL-8Rα) or CXCR2 (IL-8Rβ). These receptors differ markedly in their chemokine-binding specificity; CXCR1 only binds IL-8 and CXCL6, whereas CXCR2 can bind to multiple cytokines, including IL-8, CXCL1, and CXCL2 [4]. Ligand binding to these receptors leads to the activation of multiple primary downstream signaling pathways, including the phosphatidylinositol-3 kinase (PI3K)/Akt, phospholipase C (PLC)/protein kinase C (PKC), and mitogen-activated protein kinase (MAPK) pathways, as well as activation of focal-adhesion kinase (FAK), Rho-family GTPases, and Janus kinase (JAK)/signal transducer and activator of transcription (STAT) signaling [5,6].

In normal physiology, macrophages, endothelial cells, and epithelial cells produce IL-8 in response to infection or tissue injury, where one of the functions of IL-8 is to induce chemotaxis of granulocytes, primarily neutrophils, to the affected site. Once localized to the site of insult, IL-8 can promote resolution of infection by inducing phagocytosis, oxidative burst, and the release of DNA webs known as neutrophil extracellular traps that trap and kill invading microbes [7]. The second function of IL-8 is to activate the angiogenic response. IL-8 signaling in vascular endothelial cells induces cell proliferation, survival, and migration [8], which ultimately culminate in the formation of new blood vessels [9]. In this manner IL-8 serves to both resolve the inflammatory stimulus, and promote healing.

## 3. The IL-8/IL-8R Axis in the Tumor Microenvironment

Tumor cells can acquire the expression of various chemokine(s) and/or receptor(s) to exploit these signaling pathways for their own growth and survival. Co-opting the IL-8/IL-8R axis is now known to be an established occurrence in human cancer, and has been shown to promote tumor progression by multiple means. IL-8 expression has been detected in numerous cancer types, including solid tumors (brain, breast, cervical, colon, gastric, lung, melanoma, mesothelioma, ovarian, prostate, renal, and thyroid) and hematological malignancies (AML, CLL, Hodgkin’s lymphoma) [10]. Furthermore, a direct link between high serum IL-8 expression and disease progression has been reported in clinical studies of breast, colon, ovarian, and prostate cancers, as well as in melanoma [10]. Functional studies have revealed that tumor-derived IL-8 can function in a paracrine manner to alter the composition of immune cells within the tumor microenvironment (TME), and also in an autocrine fashion to facilitate oncogenic signaling, angiogenesis, and pro-metastatic qualities like invasion and resistance [6].

**Figure 1:**
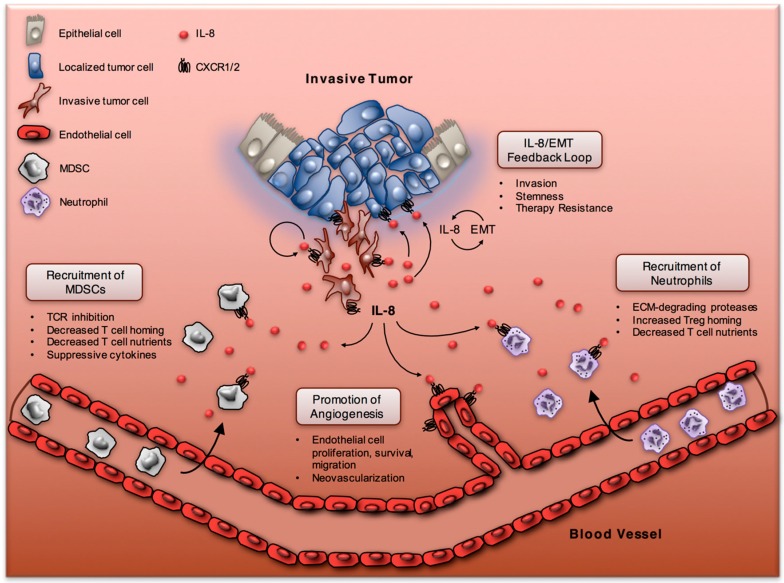
Effects of IL-8 on the tumor and microenvironment. Depiction of IL-8 promoting autocrine and paracrine tumor cell EMT, enhancing angiogenesis, and remodeling the tumor microenvironment through attraction of neutrophils and myeloid-derived suppressor cells (MDSCs).

The TME plays a pivotal role in the development and progression of cancer, and is also a major factor in governing the efficacy of a given therapeutic intervention [11]. Beyond tumor cells, the TME is composed of a multiplicity of host-derived cell types, including fibroblasts, endothelial cells, pericytes, and a great variety of infiltrating leukocytes [12]. These infiltrating immune cells may hinder tumor development by activating and engaging tumor-reactive cytotoxic mechanisms, or exert immunosuppressive functions that favor tumor growth and progression [13]. Recent advances now suggest that tumor-derived IL-8 can bias the TME into an immunosuppressive state by increasing the infiltration of neutrophils and myeloid-derived suppressor cells (MDSC, Figure 1), two related innate immune cell types with some shared phenotypic and functional properties [14].

### 3.1. IL-8 and Neutrophils

Neutrophils are the most abundant leukocytes in human peripheral blood, accounting for 50%–70% of white blood cells [15]. Although short-lived, they are essential cells of the innate immune system, and are the “first responders” during the acute phase of inflammation. They are best known for their role in killing invading microorganisms, such as bacteria and fungi, through phagocytosis and the production of activating cytokines, reactive oxygen species, and defensins. In contrast to their well-described role in microbial defense, the role of neutrophils in tumor biology has been relatively unappreciated. Neutrophils can make up a sizeable portion of the immune cell component of tumors, and so-called tumor-associated neutrophils (TAN) have been associated with worse clinical outcome in most studies of human cancers, including bronchioloalveolar adenocarcinoma [16], colorectal carcinoma [17], esophageal squamous cell carcinoma [18], head and neck squamous cell carcinoma [19], hepatocellular carcinoma [20], melanoma [21], and renal cell carcinoma [22,23]. Additionally, neutrophil infiltration correlates with tumor grade in human gliomas [24] and aggressive types of pancreatic cancer [25]. Importantly, tumor cells themselves can promote neutrophil recruitment to the TME [26], and IL-8, or its murine functional homologs CXCL1 (KC) and CXCL2 (MIP-2), have been shown to attract neutrophils to the TME in preclinical models of Ras-driven cancer [27,28,29].

Exactly how neutrophils affect the tumor is an area of current investigation, and recent evidence for plasticity of TAN phenotype suggests that they can be polarized into either antitumor N1 or pro-tumor N2 phenotypes similar to that of tumor-associated macrophages [15]. N1 TAN can directly kill tumor cells via antibody-dependent cellular cytotoxicity (ADCC) [30] or oxidative damage [31], although neutrophil-derived ROS may be mutagenic and contribute to genomic instability [32,33,34]. N1 TAN can also promote adaptive antitumor immune responses through the secretion of T cell-attracting cytokines (CCL-3, CXCL9, CXCL10), inflammatory cytokines (IL-12, TNF-α, GM-CSF) [35,36], and cross-presenting antigens to CD8+ cytotoxic T lymphocytes (CTL) [37]. In contrast, N2 TAN activate tumor angiogenesis through the production of MMP-9 [38] and facilitate metastasis by secreting the extracellular matrix-degrading enzymes collagenase-IV, heparanase, and neutrophil elastase [39,40]. Furthermore, N2 TAN can suppress adaptive immunity by starving T cells of arginine via secretion of arginase 1 [41,42], and by recruiting T regulatory cells (Tregs) through production of CCL17 [43]. TAN plasticity has been associated with tumor progression; early-stage tumors exhibited neutrophils with a more cytotoxic phenotype capable of stimulating antitumor T-cell responses [44,45], whereas neutrophils from established tumors downregulated these functions in favor of acquiring pro-tumor qualities [44]. A key determining factor seems to be signaling by TGF-β, as blockade of TGF-βRI with the small molecule SM16 was found to induce an N2-to-N1 phenotype conversion in murine mesothelioma tumors [35]. Collectively, these studies lend credence to the notion that tumor-derived IL-8 can actively suppress antitumor immunity through the recruitment of N2 neutrophils.

### 3.2. IL-8 and Myeloid-Derived Suppressor Cells

Cells of the myeloid lineage are formed in the bone marrow from myeloid progenitor cells that then migrate to peripheral organs where they differentiate into macrophages, granulocytes, or dendritic cells. However, in pathological conditions, the maturation of these cells can become arrested resulting in the production and activation of a heterogeneous population of immature immunosuppressive cells known as myeloid-derived suppressor cells (MDSCs) [46]. Two subpopulations of MDSCs are found in humans and are defined as granulocytic (CD33+ CD11b+ HLA-DR-/low CD15+) or monocytic (CD33+ CD11b+ HLA-DR-/low CD14+) [47]. Cancer patients present with increased circulating MDSCs relative to healthy controls, and the level of circulating MDSCs correlates with clinical stage [48].

Tumor cells promote the expansion of MDSCs within the TME through the production of a variety of soluble factors (including prostaglandins, GM-CSF, IL-6, and VEGF) that persistently activate JAK2/STAT3 signaling in myeloid progenitor cells [46]. MDSCs can then become activated by other factors (including IFN-γ, TGF-β, IL-4, and IL-13) that are produced by activated T cells and intra-tumoral stromal cells [46]. MDSCs suppress anti-tumor immune responses primarily by inhibiting T cells via multiple mechanisms, including (1) production of reactive oxygen and nitrogen species, which can inactivate T cell receptors [49] and CTL-attracting chemokines [50]; (2) depletion of T cell nutrients from the TME (l-arginine, l-tryptophan, and l-cysteine), which inhibit T-cell activation and proliferation [41,51,52,53]; (3) ADAM-17-mediated disruption of T-cell homing [54]; (4) production of the immunosuppressive factors TGF-β and IL-10 [55]; and (5) induction of Tregs [56].

Obtaining experimental evidence for the role of IL-8 in attracting MDSCs to the TME has been complicated by the lack of a CXCL8 gene in the rodent genome. The significance of murine functional homologs of IL-8 and CXCR2 in mediating MDSC homing in cancer, however, has been described [57]. To study human IL-8, Asfaha and colleagues developed a transgenic mouse that carriers the entire human CXCL8 gene and associated regulatory elements encoded within a bacterial artificial chromosome [58]. Using this model system, they showed that IL-8 significantly accelerates inflammation-associated colonic and gastric carcinogenesis through increased recruitment of MDSCs. Further evidence was reported in a clinical study of prostate cancer patients that found rising levels of circulating MDSCs associated with increasing stage, increasing serum IL-8 and IL-6 levels, and defective T-cell function [59]. Finally, a very recent report detailed the use of hydrodynamic gene transfer to insert the human CXCL8 gene into mouse livers, where chemoattraction of MDSCs was observed into these IL-8-producing livers [60]. This study also showed that IL-8 in vitro attracted CXCR1- and CXCR2-expressing MDSCs isolated from cancer patient peripheral blood mononuclear cells, and that IL-8 induced extrusion of neutrophil extracellular traps from MDSCs with a granulocytic phenotype.

## 4. IL-8 and Epithelial-Mesenchymal Transition

The studies referenced above present a portrait of IL-8 as an inflammatory mediator capable of remodeling the TME by recruiting suppressive neutrophils and MDSCs to dampen antitumor immunity. In addition to modulating immune cell chemotaxis, IL-8 also has potent effects on tumor cells (Figure 1). These effects can be either antitumorigenic, as is the case with CXCR2 signaling that has been shown to facilitate senescence induced by oncogene activation or by replicative exhaustion [61,62], or protumorigenic, for example via activation of the EMT differentiation program, as described below.

EMT is the process whereby epithelial cells lose their characteristic epithelial features (such as apical-basolateral polarity, extensive intercellular adhesions, and contact growth inhibition) in favor of acquiring mesenchymal features (such as leading edge-trailing edge asymmetry, loose intercellular contacts, and motility/invasiveness) [63]. This process, and the reverse process of mesenchymal-epithelial transition (MET), are well known to occur during development (Type I EMT) and tissue regeneration (Type II EMT) as cells reversibly transition between epithelial and mesenchymal phenotypes [63]. Similar observations have been made in cancer cells (Type III EMT) [63]; however, tumor cells can often acquire mesenchymal features without losing certain epithelial characteristics [64]. This so-called “metastable” or “hybrid” phenotype indicates that cell phenotype is highly plastic in carcinoma cells, and therefore may be able to respond dynamically to changing conditions of the surrounding environment [65]. Cells that have undergone acquisition of mesenchymal features (i.e., “mesenchymalization”) gain not only increased motility and invasiveness, but may also be endowed with enhanced cancer stem-like properties and greater resistance to cytotoxicity.

Given the significance of EMT in tumor pathophysiology, much attention has been focused upon elucidating the mechanisms that may trigger changes in tumor cell phenotype. A relatively small suite of transcription factors controls EMT transiently during embryogenesis or wound healing, but these factors can become aberrantly overexpressed in pathological conditions such as fibrosis and cancer. Some well-characterized examples of EMT transcription factors include the zinc-finger proteins Snail, Slug, ZEB1, and ZEB2, and the helix-loop-helix transcription factors Twist1 and Twist2 [63]. These transcription factors are, in turn, regulated by intrinsic signaling events, such as signaling by oncogenic K-Ras mutation [66], or by signaling mediated by a variety of external stimuli found within the tumor microenvironment. Examples include extracellular matrix components (collagen, hyaluronic acid), soluble growth factors (TGF-β, FGF, EGF, HGF, Wnts), cytokines (IL-6, IL-1β, TNFα), and hypoxia [67,68,69]. The source(s) of these soluble factors may include supporting non-tumor cells such as cancer-associated fibroblasts, endothelial cells, and infiltrating immune cells, or the tumor cells themselves.

### 4.1. The EMT and IL-8 Positive Feedback Loop

Once an epithelial tumor cell has gone through an EMT, it will often begin to produce the very same cytokines and/or growth factors that promote EMT. These autocrine feedback loops serve to maintain these cells in a mesenchymal state, and also induce neighboring cells to undergo EMT. For example, TGF-β is a potent EMT-inducing growth factor that promotes its own expression [70], and prolonged treatment with TGF-β in Madin-Darby canine kidney cells was shown to activate both EMT and an autocrine TGF-β signaling loop that was necessary to maintain these cells in a mesenchymal state [71]. Other examples of auto-regulatory EMT-inducing factors include IL-6 [72] and VEGF [73].

Interestingly, IL-8 also appears to share this auto-regulatory quality. EMT induction via multiple means has been shown to promote IL-8 in numerous experimental systems. For example, treatment of colon cancer cells with TNF-α and TGF-β upregulated IL-8 expression [74], as did treatment of breast cancer cells with EGF [75]. Aberrant nuclear localization of the tight junction protein ZO-1 associated with EMT and enhanced IL-8 expression [76]. Overexpression of Snail increased IL-8 secretion in vivo [77], and Snail was subsequently found to directly upregulate IL-8 by binding E-boxes within the CXCL8 gene promoter to activate transcription [78]. Conversely, treatment of tumor cells with IL-8 is known to promote EMT; IL-8 induces EMT in colon cancer cells [79], nasopharyngeal carcinoma cells [80], and breast cancer cells [81].

Another EMT regulator that has been investigated in the authors’ laboratory in connection with IL-8 is the T-box transcription factor brachyury (gene name T), which regulates the formation of the embryonic mesoderm [82]. Consistent with its role in development, brachyury has been shown to be a novel driver of EMT in human carcinomas by inducing a loss of epithelial features, a gain of mesenchymal features, and a gain of motility and invasiveness [83]. In recent years, Fernando and colleagues investigated differences in the secretory phenotype of control cells compared to those that had undergone mesenchymalization via brachyury overexpression. They found that overexpression of brachyury resulted in the enhanced secretion of various cytokines, chemokines, and angiogenic factors, including IL-6, CXCL1, CCL5, osteoprotegerin, angiogenin, VEGF, and PlGF [84]. One of the most upregulated factors was IL-8, which increased several-fold along with both of its receptors [84]. Blockade of these receptors in mesenchymalized breast cancer cells significantly reduced fibronectin expression and invasiveness, indicating that IL-8 signaling is necessary for tumor cell EMT [84]. Furthermore, addition of IL-8 upregulated brachyury expression, and IL-8/IL-8R inhibition reduced brachyury expression, demonstrating the existence of a functional brachyury/IL-8 positive feedback loop that maintains the mesenchymal phenotype [84].

### 4.2. IL-8 and Cancer Stem Cells

Cancer stem cells (CSCs) are a population of tumor cells thought to be responsible for tumor initiation, maintenance, and metastasis. They exhibit distinct functional properties, including the ability to initiate and propagate tumors when serially passaged in immunocompromised mice, and the ability to both self-renew and differentiate into non-self-renewing cells [85]. EMT is a well-documented means of acquiring CSC properties in tumor cells [86]. Uncovering methods of preferentially targeting CSCs is an area of intense research, and several studies have shown the influence of IL-8 in driving the CSC phenotype. For example, breast CSCs exhibit enhanced CXCR1 expression and increased metastasis formation [87]. IL-8 levels in the metastatic fluid from breast cancer patient samples were associated with ex vivo mammosphere formation [88], and recombinant IL-8 enhanced the CSC population, mammophere formation, and CSC invasion in vitro [87]. Blockade of CXCR1 was found to selectively deplete breast CSCs and induce FasL expression, which induced bystander killing of the non-CSC population [89]. Additionally, CXCR1 blockade in vivo decreased metastasis formation, and combination with docetaxel [89] or paclitaxel [90] reduced primary tumor growth or brain metastases, respectively, better than either agent alone.

Breast cancer is not the only malignancy in which the IL-8/IL-8R axis is associated with CSC properties. IL-8 inhibition reduced stemness marker expression, colonosphere formation, and tumor growth in colon cancer cell lines [78]. IL-8 increased tumorsphere formation, CSC populations, and cell invasion in pancreatic cancer cells, and these effects were reversed via CXCR1 blockade [91]. In acute myeloid leukemia, IL-8 and CXCR2 were found to be overexpressed in stem and progenitor cells, and targeting CXCR2 reduced the viability of leukemic stem cells [92]. Likewise, IL-8 was reported to induce stemness and EGFR inhibitor resistance in lung cancer [93], and CXCR1/2 inhibition decreased tumor growth, metastasis, and angiogenesis of lung cancer xenografts [94]. Collectively, these studies demonstrate that IL-8-driven cancer stemness appears to be a common mechanism that is conserved across multiple tumor types, and, importantly, that this mechanism is amenable to therapeutic intervention via CXCR1/2 inhibition.

### 4.3. IL-8 and EMT-Associated Resistance to Immune Killing

EMT is not only associated with metastasis and stemness, but also with resistance to various death-inducing stimuli [95,96], including cancer therapies such as chemotherapy, radiation, and certain small molecule kinase inhibitors [97,98,99,100]. This resistance often manifests as recurrence after treatment in both animal models [101,102] and human cancer patients [86]. Numerous reports have shown that IL-8 confers resistance to various chemotherapies, including platinum-based drugs [103,104,105], and 5-fluorouracil [78]. IL-8 has also been shown to grant resistance to gefitinib for EGFR-mutant lung cancer cells [93]. Mechanistic analyses determined that IL-8 enhanced cell survival by activating the PI3K/Akt and NF-κB pathways [93,105], as well as by enhancing drug efflux and anti-apoptotic gene expression [103].

An emerging concept in the EMT field is that mesenchymalization may also confer resistance to anti-tumor immune responses resulting in escape from immune destruction. A study by Kudo-Saito and colleagues [106] documented the role of EMT in immune evasion by showing that overexpression of the EMT driver Snail in melanoma cells induced the secretion of thrombospondin-1 and TGF-β, which, in turn, converted CD4+ T cells into immunosuppressive Tregs. Snail overexpressing cells were also shown to be more resistant to cytolysis by antigen-specific CTLs versus control cells. Subsequently, Akalay and colleagues demonstrated that (1) EMT induction via Snail overexpression or acquired TNF-α-resistance protects against CTL lysis by impairing immunological synapse formation and by activating autophagy [107], and (2) TGF-β inhibition in cells that have been transitioned through EMT by WISP2 knockdown restores sensitivity to CTL lysis by suppressing the stemness transcription factor Kruppel-like 4 (KLF4) [108].

Research performed in the authors’ laboratory and others’ has identified a novel potential link between IL-8 and intrinsic resistance to antitumor immunity. Brachyury, a known inducer of IL-8 and CXCR1 expression [84], was recently shown to enhance resistance to chemotherapy and radiation [109,110]. Follow-up studies demonstrated that brachyury also protects cells against apoptosis triggered by immune effector cytotoxic mechanisms [111,112]. Cytotoxic immune cells employ both caspase-dependent (FasL, TRAIL) and caspase-independent (perforin/granzyme) mechanisms to lyse target cells, and brachyury was found to mediate resistance to both mechanisms: (1) brachyury destabilizes the cell cycle regulator CDK1 thereby preventing nuclear lamin phosphorylation and subsequent degradation of the nucleus by effector caspases [111]; and (2) brachyury upregulates the C-terminal subunit of mucin 1 (MUC1-C), an oncoprotein that prevents mitochondrial permeabilization in response to caspase-dependent and -independent apoptotic stimuli [112]. Importantly, MUC1-C was shown to activate stemness in breast cancer cells via NF-κB-dependent upregulation of both IL-8 and CXCR1 expression [113]. It is tempting to speculate that an autoregulatory feedback loop may be in place in which TME-derived IL-8 initially induces tumor cell EMT and brachyury expression, which in turn upregulates MUC1-C, the NF-κB pathway, and finally IL-8 and CXCR1 expression. Once produced by the tumor cells, IL-8 could continuously induce its own expression and continuously perpetuate brachyury-mediated resistance to immune killing. Although this model is still theoretical, experiments are currently underway to address this hypothesis.

## 5. IL-8-Targeting Immunotherapy

The increasing data implicating the role of IL-8 in modulating chemotaxis, EMT, resistance, and metastasis poses a need for novel interventions to manipulate IL-8/IL-8R signaling. Small molecule inhibitors targeting CXCR1 and CXCR2 are an appealing therapeutic approach to target this pathway. Reparixin (also known as repertaxin) is a non-competitive inhibitor of CXCR1/2 that has been characterized in the context of inflammatory responses elicited by increased IL-8 signaling [114]. This small molecule inhibitor prevents IL-8 from binding and interacting with its receptors by keeping both CXCR1 and CXCR2 in an inactive conformation [115]. In preclinical studies, reparixin has been shown to deplete CSC tumor cells in vitro and reduce tumor growth in vivo [89]. Interestingly, reparixin has also been combined with chemotherapy, and demonstrated a significant combinatorial effect as evidenced by reduced tumor metastases, tumor debulking, and reduction of CSC populations in triple negative breast cancer cell lines and xenografts [90]. Reparixin has been used in Phase II clinical trials in pancreatic islet transplantation [114] and delayed graft dysfunction after kidney transplantation [116], as well as in a Phase I clinical trial in combination with paclitaxel for the treatment of metastatic triple negative breast cancer [117]. These trials substantiate the potential for IL-8 inhibition to be utilized in the clinic as a single agent or in combination with chemotherapeutic agents. In addition to reparixin, several other small molecule inhibitors that block CXCR2 signaling have been evaluated preclinically and/or clinically for various pathological conditions (reviewed in [118]).

IL-8 neutralizing antibodies have also been developed to block IL-8 signaling, two of which have been studied extensively: ABX-IL8 and HuMax-IL8 (previously HuMab10F8). In preclinical studies, neutralization of IL-8 via ABX-IL8 was shown to reduce invasion and angiogenesis in melanoma tumor-bearing mice by inhibition of matrix metalloproteinase-2 and increased tumor cell apoptosis [119]. In addition, HuMax-IL8 was previously shown to decrease IL-8-induced neutrophil activation and migration in vitro, as well as beneficial anti-inflammatory responses in patients with palmoplantar pustulosis (PPP) [120]. Patients with PPP tolerated HuMax-IL8 well, and this antibody is currently being used in a Phase I clinical trial in advanced malignant solid tumors [121]. Blocking IL-8 activity has the potential to diminish the tumor-promoting signals for angiogenesis, neutrophil and MDSC activation/infiltration, EMT, as well as reducing bulk tumor growth and metastasis.

## 6. Conclusions

Cancer treatment is in the midst of a revolution brought about by the successful development and implementation of immunotherapies. However, as with the conventional and targeted therapies that came before, resistance to immunotherapy, whether intrinsic or extrinsic, presents a significant barrier to the expansion of these approaches to the wider patient population. The collective work of many laboratories indicates that the IL-8/IL-8R axis is a common pathway to resistance for tumor cells. As a chemoattractant for neutrophils and MDSCs, IL-8 is able to condition the TME to become immunosuppressive and favor tumor progression. As an inducer of tumor cell mesenchymalization, IL-8 is able to activate stemness and anti-apoptotic processes that circumvent killing by cytotoxic immune cells. Future efforts to target the IL-8/IL-8R axis, whether alone or in combination with other immunotherapies, must be undertaken to evaluate the effectiveness of this promising strategy for overcoming tumor immunoresistance.

## Abbreviations

The following abbreviations are used in this manuscript:

CSC	cancer stem cell
CXCL	cysteine-X-cysteine ligand
CXCR	cysteine-X-cysteine chemokine receptor
CTL	cytotoxic T lymphocyte
EMT	epithelial-mesenchymal transition
FAK	focal-adhesion kinase
IL	interleukin
IL-8R	interleukin-8 receptor
JAK	Janus kinase
MAPK	mitogen-activated protein kinase
MDSC	myeloid-derived suppressor cell
MUC1-C	mucin 1 C-terminal subunit
NK	natural killer cell
PI3K	phosphatidylinositol-3 kinase
PKC	protein kinase C
PLC	phospholipase C
PPP	palmoplantar pustulosis
STAT	signal transducer and activator of transcription
Treg	T regulatory cell
TAN	tumor-associated neutrophil
TME	tumor microenvironment
